# Supplementary material for: MCL‑1 safeguards activated hair follicle stem cells to enable adult hair regeneration
Source: Nat Commun. 2025 Mar 22;16:2829. doi: 10.1038/s41467-025-58150-5 (PMC11929845; doi:10.1038/s41467-025-58150-5)
Supplement: Supplementary file 1 — Supplementary Information [file 41467_2025_58150_MOESM1_ESM.pdf]

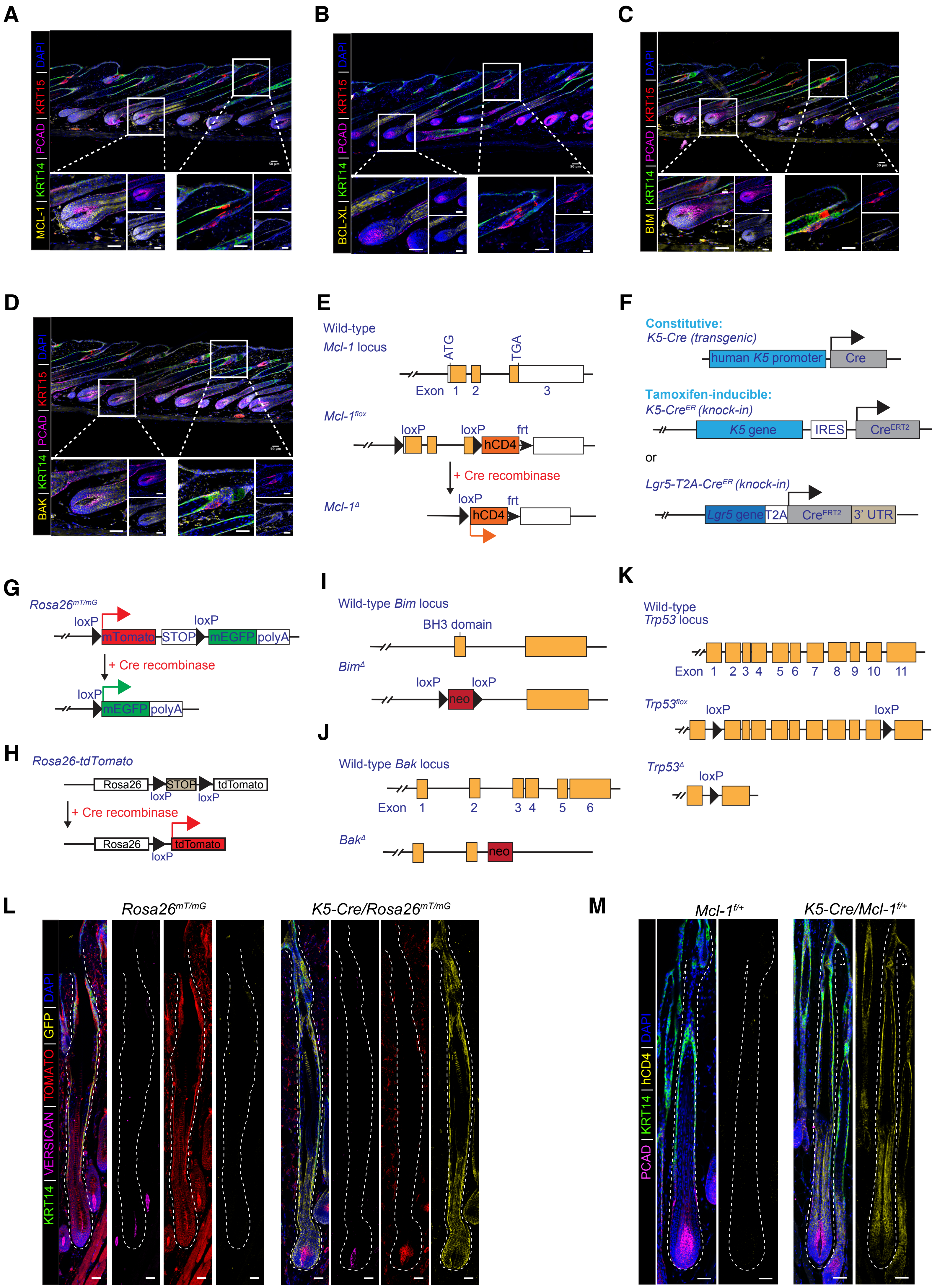

A
MCL-1 | KRT14 | PCAD | KRT15 | DAPI
B
BCL-XL | KRT14 | PCAD | KRT15 | DAPI
C
BIM | KRT14 | PCAD | KRT15 | DAPI
D
BAK | KRT14 | PCAD | KRT15 | DAPI
E
Wild-type
Mcl-1 locus
ATG
TGA
Exon 1 2 3
Mcl-1flox
loxP
loxP
frt
hCD4
+ Cre recombinase
Mcl-1Δ
loxP
frt
hCD4
F
Constitutive:
K5-Cre (transgenic)
human K5 promoter
Cre
Tamoxifen-inducible:
K5-CreER (knock-in)
K5 gene
IRES
CreERT2
or
Lgr5-T2A-CreER (knock-in)
Lgr5 gene
T2A
CreERT2
3' UTR
G
Rosa26mT/mG
loxP
mTomato
STOP
loxP
mEGFP
polyA
+ Cre recombinase
loxP
mEGFP
polyA
H
Rosa26-tdTomato
Rosa26
STOP
tdTomato
loxP
loxP
+ Cre recombinase
Rosa26
tdTomato
loxP
I
Wild-type Bim locus
BH3 domain
BimΔ
loxP
neo
loxP
J
Wild-type Bak locus
Exon 1 2 3 4 5 6
BakΔ
neo
K
Wild-type
Trp53 locus
Exon 1 2 3 4 5 6 7 8 9 10 11
Trp53flox
loxP
loxP
Trp53Δ
loxP
L
Rosa26mT/mG
K5-Cre/Rosa26mT/mG
KRT14 | VERSICAN | TOMATO | GFP | DAPI
M
Mcl-1f/+
K5-Cre/Mcl-1f/+
PCAD | KRT14 | hCD4 | DAPI

Supplementary Figure Legends

Figure S1. Genetic mouse model systems to interrogate the contribution of MCL-1 in regulating HF cell survival, hair growth, and hair regeneration

(A-D) Representative confocal images showing the expression of MCL-1 (A), BCL-XL (B), BIM (C), and BAK (D) in HFs during anagen (P29). Basal skin layer is marked by KRT14, HFSCs by KRT15, and hair matrix by PCAD, with DAPI as a nuclear counterstain (*n*=4 mice, scale bars: 50 µm). **(E)** Schematic of the floxed *Mcl-1* model, where CRE-mediated recombination deletes *Mcl-1* and activates an hCD4 reporter, indicating gene deletion and *Mcl-1* promoter activity. **(F)** K5 promoter-driven CRE models for skin-specific gene deletion. The constitutive K5-Cre model uses a 5 kb *Krt5*promoter to drive CRE expression, while the tamoxifen-inducible K5-CreERT2 model allows temporal control. The *Lgr5-T2A-CreER* model, generated by inserting a T2A-CRE-ERT2 cassette into the *Lgr5* gene, enables inducible deletion in *Lgr5*$^{+}$ HFSCs. **(G-H)** The *Rosa26*$^{mT/mG}$ CRE and *Rosa26-tdTomato* reporter models allows lineage tracing of CRE-expressing cells by switching from membrane-bound Tomato (pre-recombination) to eGFP (post-recombination) (G) or activating the expression of Tomato (H). **(I-K)** Gene knockout strategies for apoptosis regulators. *Bim* knockout (I) disrupts the BH3 domain via a neomycin resistance cassette. *Bak* knockout (J) removes exons encoding BH1-3 domains. *Trp53* floxed (K) mice contain loxP sites flanking exons 2-10, allowing CRE-mediated deletion of *Trp53*. **(L)** Validation of K5 promoter-driven CRE recombinase activity. The K5-Cre/*Rosa26*$^{mT/mG}$ model confirms high specificity and efficiency of CRE activity in the skin epithelium. Confocal images show GFP and Tomato expression, with versican labelling dermal papilla (*n*=3 mice, scale bars: 50 µm). **(M)** Confirmation of *Mcl-1* deletion using the hCD4 reporter. Confocal images show hCD4 expression in *K5-Cre/Mcl-1*$^{f/+}$ mice compared to control littermates (*n*=5 mice, scale bars: 50 µm). Basal skin is stained by KRT14, TACs by PCAD, and nuclei by DAPI.

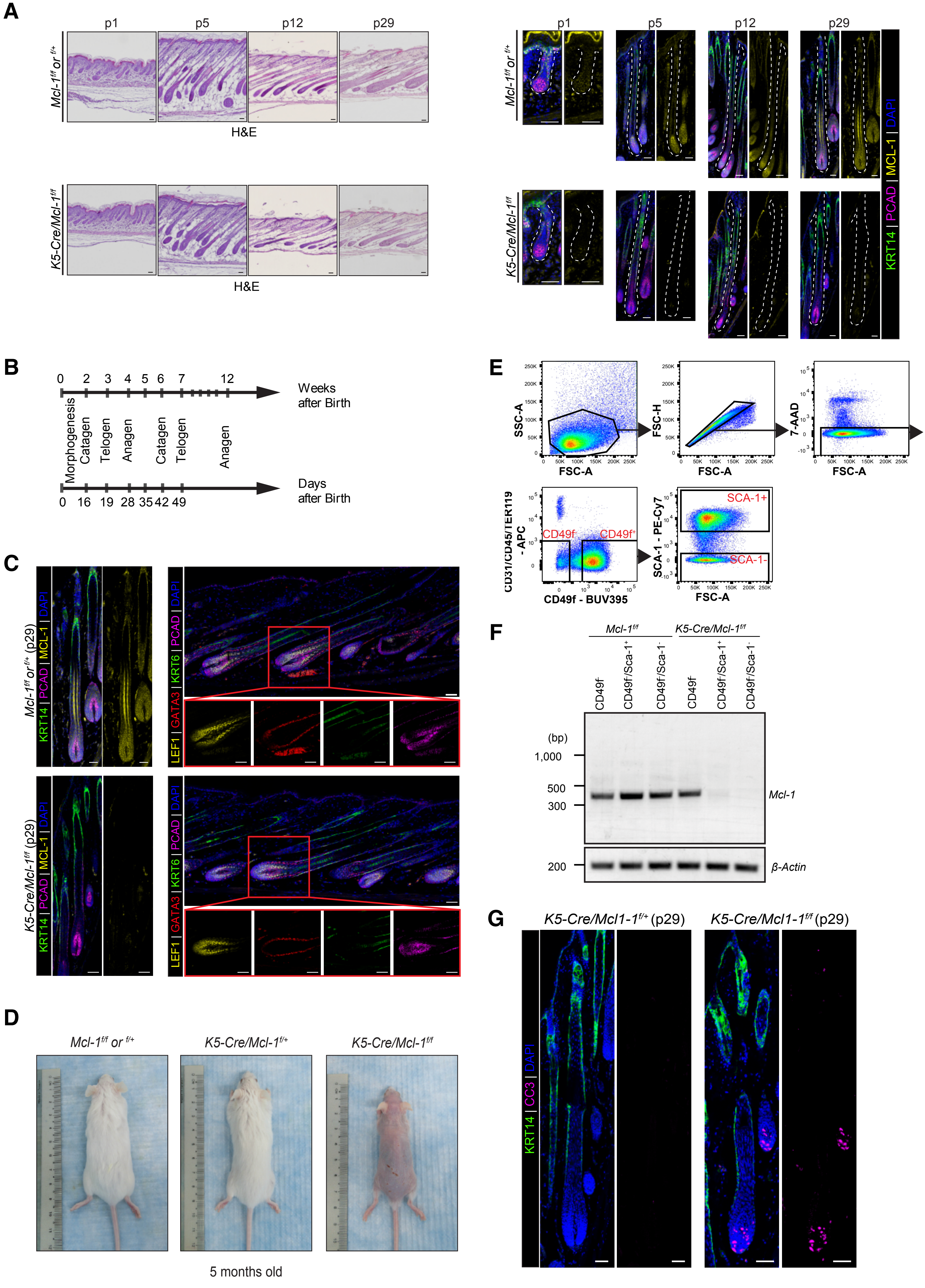

A
p1
p5
p12
p29
Mcl-1f/f or f/+
H&E
K5-Cre/Mcl-1f/f
KRT14 | PCAD | MCL-1 | DAPI
B
Weeks after Birth
Morphogenesis
Catagen
Telogen
Anagen
Catagen
Telogen
Anagen
Days after Birth
C
Mcl-1f/f or f/+ (p29)
K5-Cre/Mcl-1f/f (p29)
KRT14 | PCAD | MCL-1 | DAPI
LEF1 | GATA3 | KRT6 | PCAD | DAPI
D
Mcl-1f/f or f/+
K5-Cre/Mcl-1f/+
K5-Cre/Mcl-1f/f
5 months old
E
SSC-A
FSC-A
FSC-H
7-AAD
CD31/CD45/TER119 - APC
CD49f - BUV395
CD49f-
CD49f+
SCA-1 - PE-Cy7
SCA-1+
SCA-1-
F
Mcl-1f/f
K5-Cre/Mcl-1f/f
CD49f
CD49f/Sca-1+
CD49f/Sca-1-
(bp)
1,000
500
300
200
Mcl-1
β-Actin
G
K5-Cre/Mcl1-1f/+ (p29)
K5-Cre/Mcl1-1f/f (p29)
KRT14 | CC3 | DAPI

Figure S2: The absence of MCL-1 does not affect HF organogenesis but results in premature hair loss

(A) Representative images of H&E-stained histological sections and confocal images showing that *Mcl-1* deficiency does not impair HF morphogenesis nor early stages of HF growth up to p29. Scale bars, 50 µm. n=4 mice per genotype for each development stage. **(B)** Schematic illustration of the postnatal HF development in mice. After birth, HFs undergo morphogenesis. The first postnatal hair cycle is synchronous and occurs between p20 (first telogen stage) to p55 (second telogen stage). After that, the HF cycling only occurs in small patches. **(C)** Representative confocal images of HF matrix and cortex (LEF1), inner root sheath (GATA3), suprabasal ORS and medulla (KRT6) in *K5-Cre/Mcl-1*$^{f/f}$ mice and *Mcl-1*$^{f/f}$ or *Mcl-1*$^{f/+}$ littermate control mice. Scale bars, 50 µm. n=3 mice per genotype. **(D)** Representative photographs showing complete loss of hair in adult *Mcl-1* conditional knockout mice. *Mcl-1*$^{f/f}$ or $^{f/+}$, *K5-Cre/Mcl-1*$^{f/+}$ and *K5-Cre/Mcl-1*$^{f/f}$ mice at the age of more than 5 months. n=9 mice per genotype. **(E)** Strategy for FACS sorting of skin cell subpopulations. Representative FACS plots showing the non-epithelial cells (CD49f^{-}), HF (CD49f^{+}/SCA-1^{-}) and interfollicular epidermis (CD49f^{+}/SCA-1^{+}) cell subsets. **(F)** Representative gel images showing efficient deletion of the floxed *Mcl-1* gene in interfollicular epidermis (CD49f^{+}/SCA-1^{+}) and HF (CD49^{+}/SCA-1^{-}) cells but not in non-epidermal cells (CD49f^{-}) in *K5-Cre/Mcl-1*$^{f/f}$ mice compared to *Mcl-1*$^{f/f}$ littermates. Skin cell subsets defined by CD49f and SCA-1 expression were FACS sorted and *Mcl-1* transcripts were amplified by RT-PCR. n=2 independent experiments. **(G)** The presence of one *Mcl-1* allele is sufficient to prevent apoptosis in the skin of *K5-Cre/Mcl-1*$^{f/+}$ mice. Representative confocal image showing the absence of activated CC3^{+} cells in the skin from *K5-Cre/Mcl-1*$^{f/+}$ and *K5-Cre/Mcl-1*$^{f/f}$ mice during mid anagen (p29) (n=5 mice per genotype). Basal skin layer is stained by KRT14, hair matrix containing TACs is stained by PCAD and DAPI is used as a nuclear counterstain in each image in panel (A, C and G).

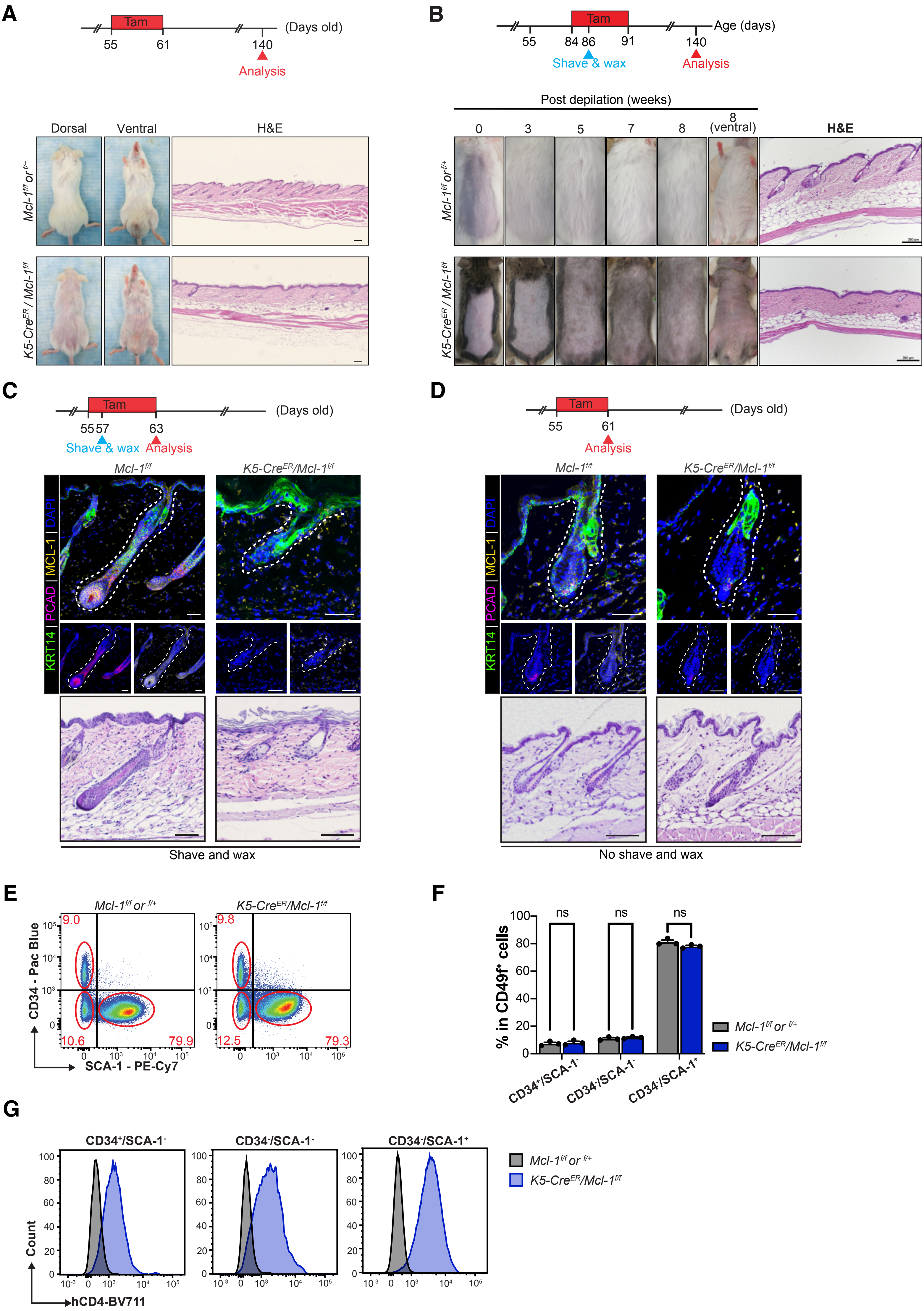
A
Tam
55
61
140
(Days old)
Analysis
Dorsal
Ventral
H&E
Mcl-1f/f or f/+
K5-CreER / Mcl-1f/f
B
Tam
55
84 86
91
140
Age (days)
Shave & wax
Analysis
Post depilation (weeks)
0
3
5
7
8
8 (ventral)
H&E
Mcl-1f/f or f/+
K5-CreER / Mcl-1f/f
C
Tam
55 57
63
(Days old)
Shave & wax
Analysis
Mcl-1f/f
K5-CreER/Mcl-1f/f
KRT14 | PCAD | MCL-1 | DAPI
Shave and wax
D
Tam
55
61
(Days old)
Analysis
Mcl-1f/f
K5-CreER/Mcl-1f/f
KRT14 | PCAD | MCL-1 | DAPI
No shave and wax
E
Mcl-1f/f or f/+
K5-CreER/Mcl-1f/f
9.0
10.6
79.9
9.8
12.5
79.3
CD34 - Pac Blue
SCA-1 - PE-Cy7
F
ns
ns
ns
% in CD49f+ cells
CD34+/SCA-1-
CD34-/SCA-1-
CD34-/SCA-1+
Mcl-1f/f or f/+
K5-CreER/Mcl-1f/f
G
CD34+/SCA-1-
CD34-/SCA-1-
CD34-/SCA-1+
Count
hCD4-BV711
Mcl-1f/f or f/+
K5-CreER/Mcl-1f/f

Figure S3: Acute deletion of the *Mcl-1* gene in adult mice does not result in the immediate loss of quiescent $CD34^+$ bulge stem cell populations

(A) Inducible deletion of *Mcl-1* during adulthood leads to progressive hair loss. Mice at P55 (second telogen) were placed on a tamoxifen-containing diet for one week to delete *Mcl-1* in Krt5-expressing cells. Mice were aged and monitored. Representative photographs and H&E-stained skin sections of K5-CreER/Mcl-1f/f and control (*Mcl-1$^{f/f}$*) mice at P140. (*n*=6 mice per genotype, scale bars: 100 µm). **(B)** *Mcl-1* deletion during an asynchronized hair cycle impairs hair regeneration. Tamoxifen-induced deletion was initiated at P84, and a portion of the dorsal skin was shaved and waxed to induce hair regeneration. Representative images of *K5-Cre^{ER}/Mcl-1$^{f/f}$* and control mice at 0, 2, 5, 7, and 8 weeks post-depilation. H&E-stained skin sections at P140. (*n*=6 mice per genotype, scale bars: 200 µm). **(C, D)** *Mcl-1* deletion results in the loss of $PCAD^+$ hair germ cells only after depilation. Mice were analyzed 6 days after tamoxifen administration with (C) or without (D) depilation. Representative H&E-stained sections and confocal images of MCL-1, KRT14, and PCAD in the dorsal skin of *K5-Cre^{ER}/Mcl-1$^{f/f}$* and control mice. (*n*=3 mice per genotype, scale bars: 50 µm). **(E, F)** FACS analysis of epithelial cell populations 6 days after tamoxifen administration. Representative FACS plots (E) and quantification (F) of $CD49f^+$ epithelial cells fractionated by CD34 and SCA-1 expression in *K5-Cre^{ER}/Mcl-1$^{f/f}$* and control mice. (*n*=8 mice per genotype, data shown as mean ± SEM, n.s. = non-significant; Student's t-test). **(G)** Efficient *Mcl-1* deletion confirmed by hCD4 reporter expression. FACS analysis showing high hCD4 expression in hair bulge cells ($CD49f^+/CD34^+/SCA\text{-}1^-$), non-bulge follicular cells ($CD49f^+/CD34^-/SCA\text{-}1^-$), and interfollicular epidermis ($CD49f^+/CD34^-/SCA\text{-}1^+$) from *K5-Cre^{ER}/Mcl-1$^{f/f}$* mice, but not in controls. (*n*=3 mice per genotype).

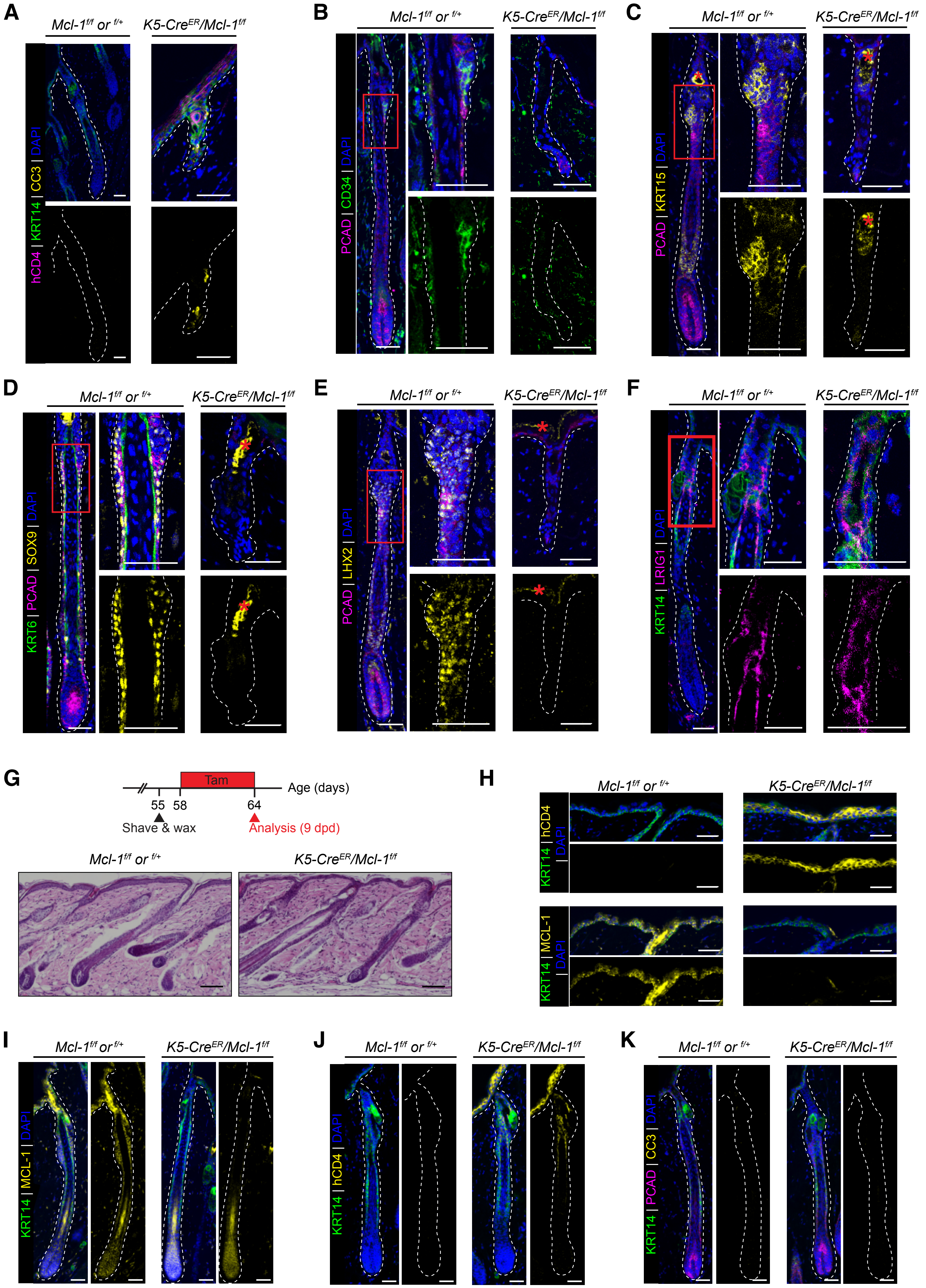

A
Mcl-1f/f or f/+
K5-CreER/Mcl-1f/f
hCD4 | KRT14 | CC3 | DAPI
B
Mcl-1f/f or f/+
K5-CreER/Mcl-1f/f
PCAD | CD34 | DAPI
C
Mcl-1f/f or f/+
K5-CreER/Mcl-1f/f
PCAD | KRT15 | DAPI
D
Mcl-1f/f or f/+
K5-CreER/Mcl-1f/f
KRT6 | PCAD | SOX9 | DAPI
E
Mcl-1f/f or f/+
K5-CreER/Mcl-1f/f
PCAD | LHX2 | DAPI
F
Mcl-1f/f or f/+
K5-CreER/Mcl-1f/f
KRT14 | LRIG1 | DAPI
G
Tam
Age (days)
55
58
64
Shave & wax
Analysis (9 dpd)
Mcl-1f/f or f/+
K5-CreER/Mcl-1f/f
H
Mcl-1f/f or f/+
K5-CreER/Mcl-1f/f
KRT14 | hCD4 | DAPI
KRT14 | MCL-1 | DAPI
I
Mcl-1f/f or f/+
K5-CreER/Mcl-1f/f
KRT14 | MCL-1 | DAPI
J
Mcl-1f/f or f/+
K5-CreER/Mcl-1f/f
KRT14 | hCD4 | DAPI
K
Mcl-1f/f or f/+
K5-CreER/Mcl-1f/f
KRT14 | PCAD | CC3 | DAPI

Figure S4: Acute deletion of Mcl-1 leads to the loss of the heterogenous pool of HFSC

(A) Representative confocal image showing increased apoptotic cells as marked by CC3 9 dpd in *Mcl-1*-deficient (hCD4$^+$) HFs of *K5-Cre*^{ER}*/Mcl-1*$^{f/f}$ mice. **(B-F)** Confocal image analyses of HFSCs as marked by CD34 (B), KRT15 (C), SOX9 (D), LHX2 (E) and LRIG1 (F) in HFs of *K5-Cre*^{ER}*/Mcl-1*$^{f/f}$ mice 9 dpd. MCL-1 (A), (n=9 mice per genotype). Scale bars: 50 µm. **(G-K)** Experimental strategy for to induce deletion of *Mcl-1* specifically in the interfollicular epidermis of adult *K5-Cre*^{ER}*/Mcl-1*$^{f/f}$ mice. A portion of the dorsal skin was shaved and waxed to induce hair regeneration, and *Mcl-1* deletion was induced 3 dpd via tamoxifen-containing diet. Dorsal skin samples were harvested 9 dpd. Representative H&E-stained histological sections (G) and confocal image analysis of MCL-1 and hCD4 (H), MCL-1 (I), hCD4 (J), and CC3 (K) expressions in the dorsal skin from *K5-Cre*^{ER}*/Mcl-1*$^{f/f}$ mice and control littermates after administration of tamoxifen diet *3* days following depilation. Scale bars, 50 µm. n=9 mice per genotype. Basal skin layer is stained by KRT14, IRS stained by KRT6, hair matrix containing TACs is stained by PCAD and DAPI is used as a nuclear counterstain where applicable in each confocal image.

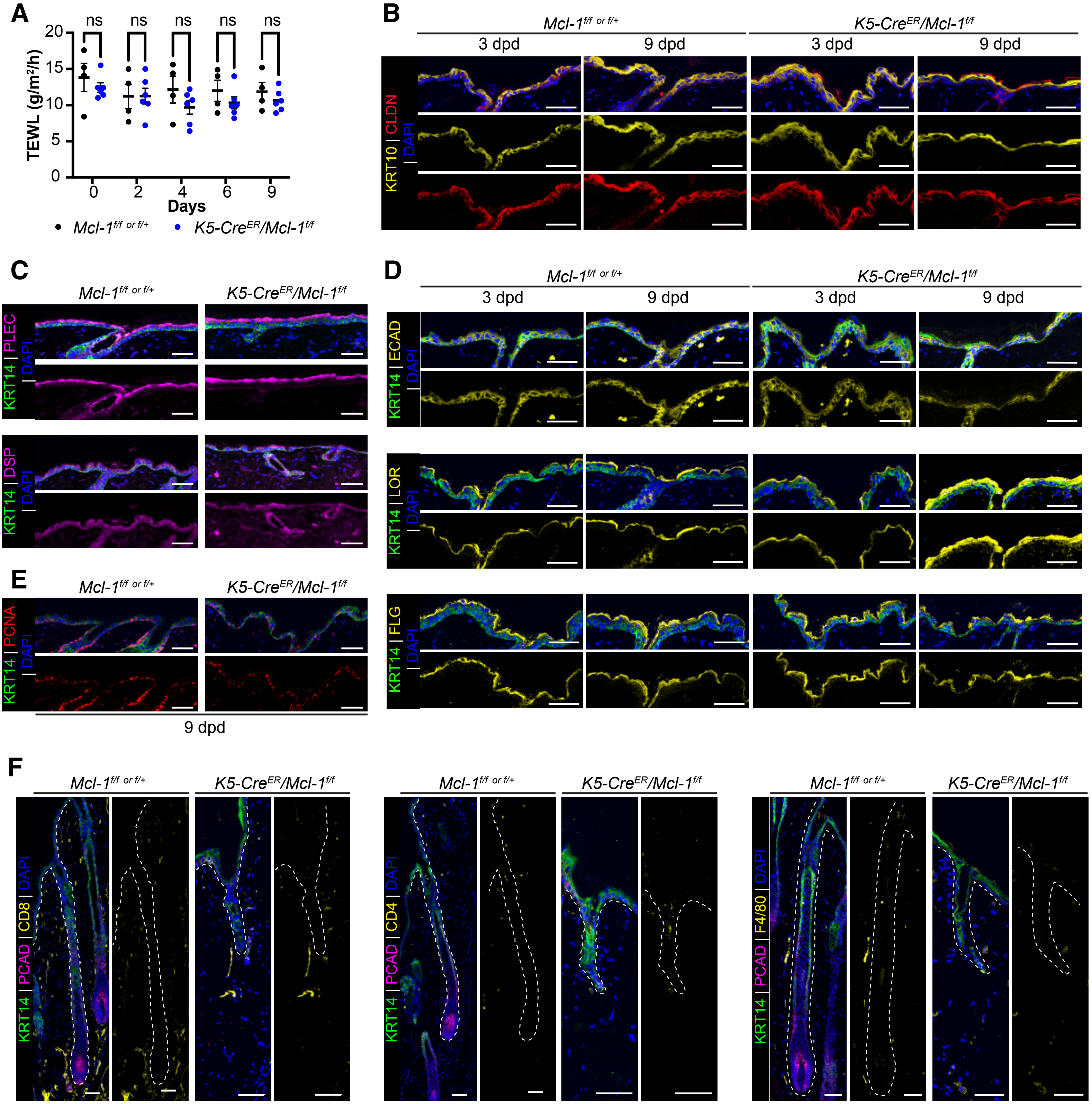

A
ns
TEWL (g/m²/h)
20
15
10
5
0
0
2
4
6
9
Days
Mcl-1f/f or f/+
K5-CreER/Mcl-1f/f
B
Mcl-1f/f or f/+
K5-CreER/Mcl-1f/f
3 dpd
9 dpd
KRT10 | CLDN | DAPI
C
Mcl-1f/f or f/+
K5-CreER/Mcl-1f/f
KRT14 | PLEC | DAPI
KRT14 | DSP | DAPI
D
Mcl-1f/f or f/+
K5-CreER/Mcl-1f/f
3 dpd
9 dpd
KRT14 | ECAD | DAPI
KRT14 | LOR | DAPI
KRT14 | FLG | DAPI
E
Mcl-1f/f or f/+
K5-CreER/Mcl-1f/f
KRT14 | PCNA | DAPI
9 dpd
F
Mcl-1f/f or f/+
K5-CreER/Mcl-1f/f
KRT14 | PCAD | CD8 | DAPI
KRT14 | PCAD | CD4 | DAPI
KRT14 | PCAD | F4/80 | DAPI

Figure S5: MCL-1 deficiency does not impact skin stratification, skin barrier and skin cell structure.

(A) Transepidermal water loss from the dorsal skin of *K5-Cre*^{ER}*/Mcl-1*$^{f/f}$ mice and control littermates were measured at 0, 2, 4, 6 and 9 days post depilation. Data are presented as mean ± SEM (n=4-5 mice per genotype). n.s. = non-significant. Student's t-test. **(B-E)** Representative confocal images showing skin stratification markers, CLAUDIN-1 (CLDN1) and KRT10 (B), skin structure markers PLECTIN (PLEC), DESMOPLAKIN (DSP) (C), skin barrier markers E-CADHERIN (ECAD), LORICRIN (LOR), and FILLAGRIN (FLG) (D), and proliferation marker PCNA (E) in dorsal skins of *K5-Cre/Mcl-1*$^{f/f}$ mice and *Mcl-1*$^{f/f}$ or *Mcl-1*$^{f/+}$ littermate control mice at 3 or 9 dpd. **(F)** Representative confocal image analysis of CD8 (T cells), CD4 (T cells), F4/80 (macrophage) in dorsal skins of *K5-Cre/Mcl-1*$^{f/f}$ mice and *Mcl-1*$^{f/f}$ or *Mcl-1*$^{f/+}$ littermate control mice at 9 dpd**.** Scale bars, 50 µm. n=9 mice per genotype. Basal skin layer is stained by KRT14, hair matrix containing TACs is stained by PCAD and DAPI is used as a nuclear counterstain where applicable in each confocal image.

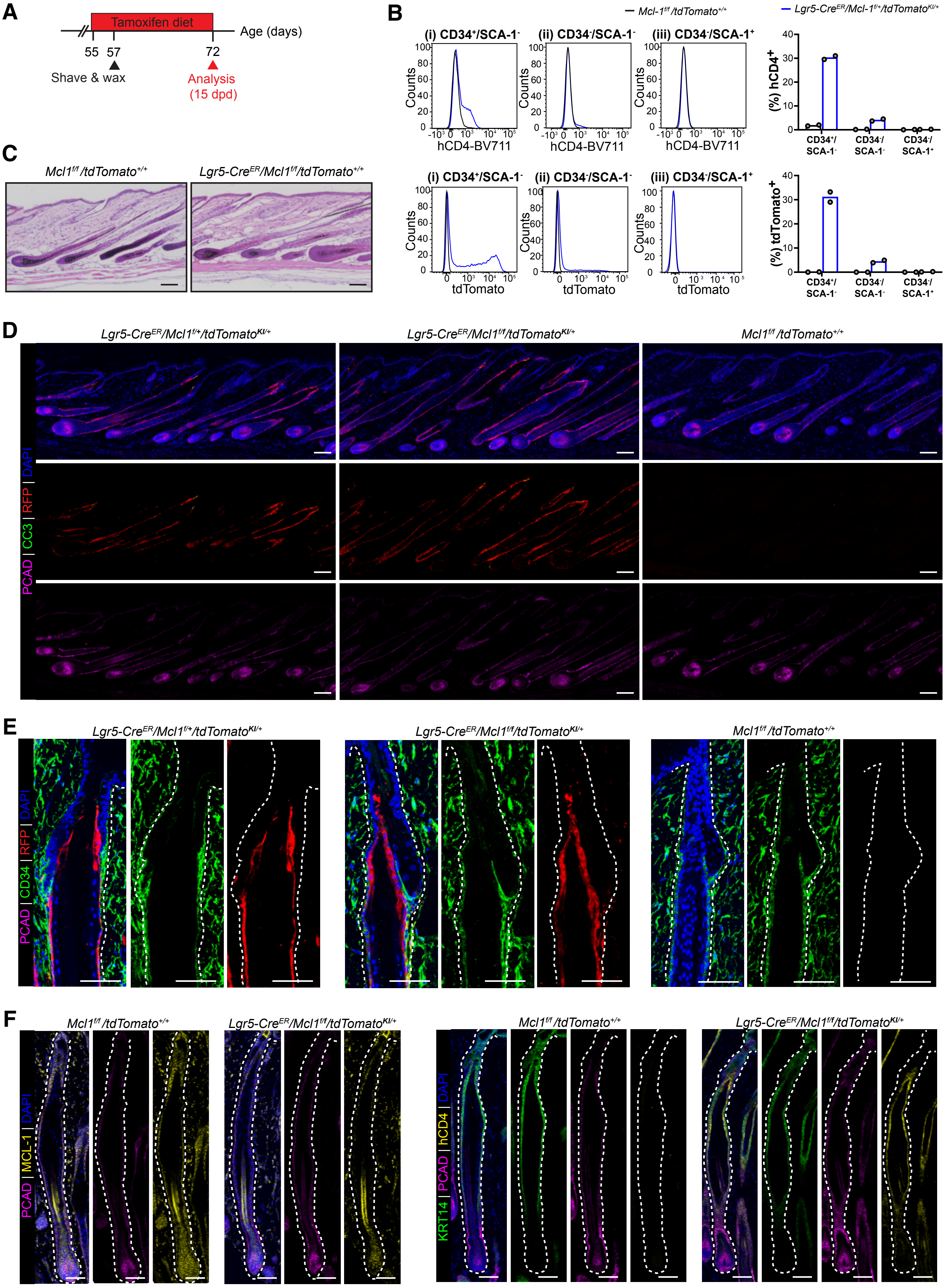

A
Tamoxifen diet
Age (days)
55
57
72
Shave & wax
Analysis
(15 dpd)
B
Mcl-1f/f /tdTomato+/+
Lgr5-CreER/Mcl-1f/+/tdTomatoKI/+
(i) CD34+/SCA-1-
(ii) CD34-/SCA-1-
(iii) CD34-/SCA-1+
Counts
hCD4-BV711
tdTomato
(%) hCD4+
(%) tdTomato+
C
Mcl1f/f /tdTomato+/+
Lgr5-CreER/Mcl1f/f/tdTomato+/+
D
Lgr5-CreER/Mcl1f/+/tdTomatoKI/+
Lgr5-CreER/Mcl1f/f/tdTomatoKI/+
Mcl1f/f /tdTomato+/+
PCAD | CC3 | RFP | DAPI
E
Lgr5-CreER/Mcl1f/+/tdTomatoKI/+
Lgr5-CreER/Mcl1f/f/tdTomatoKI/+
Mcl1f/f /tdTomato+/+
PCAD | CD34 | RFP | DAPI
F
Mcl1f/f /tdTomato+/+
Lgr5-CreER/Mcl1f/f/tdTomatoKI/+
PCAD | MCL-1 | DAPI
KRT14 | PCAD | hCD4 | DAPI

Figure S6: *Mcl-1* deletion in *Lgr5*$^{+}$ HFSCs does not impair hair regeneration in adult mice. (A) Mice at p55 (the second telogen phase) were placed on tamoxifen containing diet throughout the experiment (from p55-p72) to induce *Mcl-1* deletion in *Lgr5*-expressing HFSCs and their progeny. Dorsal skin was shaved and waxed at p57 to induce hair regeneration and subsequently harvested at 15 days post depilation (dpd). **(B)** Representative FACS plots showing the expression of tdTomato and hCD4, as markers of Cre-mediated recombination and *Mcl-1* deletion, in different subsets of HF cells. Bar graph shows mean from n=2 mice for indicated genotypes. **(C)** Representative images of H&E-stained histological sections of the skin from *Lgr5-Cre*^{ER}*/Mcl-1*$^{f/f}$*/tdTomato*$^{+/+}$ (or *Lgr5-Cre*^{ER}*/Mcl-1*$^{f/f}$*/tdTomato*$^{KI/+}$) and *Mcl-1*$^{f/f}$*/tdTomato*$^{+/+}$ (or *Mcl-1*$^{f/f}$*/tdTomato*$^{KI/+}$) control mice at 15 dpd. Scale bars, 100 µm. n=3 mice per genotype. **(D)** Representative confocal images showing cleaved caspase 3 (CC3), tdTomato^{+} (RFP), PCAD in skin sections of *Lgr5-Cre*^{ER}*/Mcl-1*$^{f/f}$*/tdTomato*$^{KI/+}$, *Lgr5-Cre*^{ER}*/Mcl-1*$^{f/+}$*/tdTomato*$^{KI/+}$ and *Mcl-1*$^{f/f}$*/tdTomato*$^{+/+}$ mice at 15 dpd. Tissue sections were counterstained with DAPI. Scale bars, 100 µm. n=4 mice per genotype. **(E)** Representative confocal images of tdTomato (RFP) and HFSCs as marked by CD34 in HFs of *Lgr5-Cre*^{ER}*/Mcl-1*$^{f/f}$*/tdTomato*$^{KI/+}$, *Lgr5-Cre*^{ER}*/Mcl-1*$^{f/+}$*/tdTomato*$^{KI/+}$ and *Mcl-1*$^{f/f}$*/tdTomato*$^{+/+}$ mice at 15 dpd. Tissue sections were counterstained with DAPI. Scale bars, 100 µm. n=4 mice per genotype. **(F-G)** Representative confocal images of MCL-1 (F), hCD4 (G), PCAD and KRT14 in HFs of *Lgr5-Cre*^{ER}*/Mcl-1*$^{f/f}$*/tdTomato*$^{KI/+}$ and *Mcl-1*$^{f/f}$*/tdTomato*$^{+/+}$ mice at 15 dpd. Cell nuclei were counterstained with DAPI. Scale bars, 50 µm. n=4 mice per genotype.

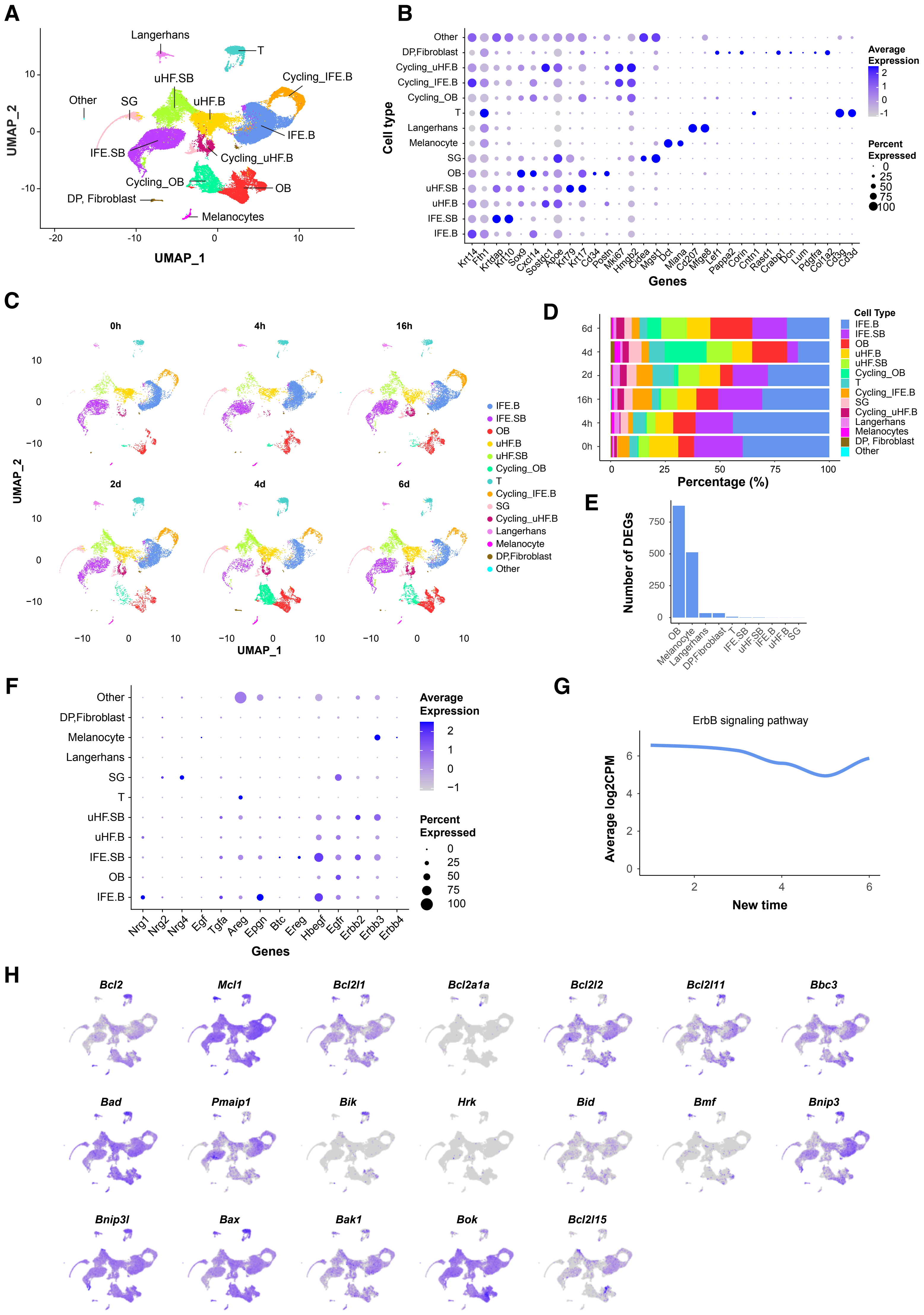
A
Langerhans
T
uHF.SB
Cycling_IFE.B
Other
SG
uHF.B
IFE.B
IFE.SB
Cycling_uHF.B
Cycling_OB
OB
DP, Fibroblast
Melanocytes
UMAP_2
UMAP_1
B
Cell type
Other
DP,Fibroblast
Cycling_uHF.B
Cycling_IFE.B
Cycling_OB
T
Langerhans
Melanocyte
SG
OB
uHF.SB
uHF.B
IFE.SB
IFE.B
Krt14 Fth1 Krtdap Krt10 Sox9 Cxcl14 Sostdc1 Apoe Krt79 Krt17 Cd34 Postn Mki67 Hmgb2 Cidea Mgst1 Dct Mlana Cd207 Mfge8 Lef1 Pappa2 Corin Cntn1 Rasd1 Crabp1 Dcn Lum Pdgfra Col1a2 Cd3g Cd3d
Genes
Average Expression
Percent Expressed
C
0h
4h
16h
2d
4d
6d
UMAP_2
UMAP_1
IFE.B
IFE.SB
OB
uHF.B
uHF.SB
Cycling_OB
T
Cycling_IFE.B
SG
Cycling_uHF.B
Langerhans
Melanocyte
DP,Fibroblast
Other
D
6d
4d
2d
16h
4h
0h
Percentage (%)
Cell Type
IFE.B
IFE.SB
OB
uHF.B
uHF.SB
Cycling_OB
T
Cycling_IFE.B
SG
Cycling_uHF.B
Langerhans
Melanocytes
DP, Fibroblast
Other
E
Number of DEGs
OB
Melanocyte
Langerhans
DP,Fibroblast
T
IFE.SB
uHF.SB
IFE.B
uHF.B
SG
F
Other
DP,Fibroblast
Melanocyte
Langerhans
SG
T
uHF.SB
uHF.B
IFE.SB
OB
IFE.B
Nrg1 Nrg2 Nrg4 Egf Tgfa Areg Epgn Btc Ereg Hbegf Egfr Erbb2 Erbb3 Erbb4
Genes
Average Expression
Percent Expressed
G
ErbB signaling pathway
Average log2CPM
New time
H
Bcl2
Mcl1
Bcl2l1
Bcl2a1a
Bcl2l2
Bcl2l11
Bbc3
Bad
Pmaip1
Bik
Hrk
Bid
Bmf
Bnip3
Bnip3l
Bax
Bak1
Bok
Bcl2l15

Figure S7. Comprehensive single-cell transcriptome atlas of murine dorsal skin at early depilation-induced anagen

(A) UMAP visualization of all cells in the murine dorsal skin across six time points at the early anagen stages of hair regeneration after depilation, colored according to the main cell classes. The plot was generated by integration of 6343, 6955, 7806, 8359, 10740 and 8574 cells for 0 h, 4 h, 16 h, 2 d, 4 d and 6 d post depilation, respectively. **(B)** Dot plot of marker genes used for annotation of cell clusters in **(A)**. The colour scales correspond to the averaged expression levels. The dot size corresponds to the ratio of cells expressing the gene in the cell type. **(C)** UMAP visualization of all cells in the dorsal skin sample for each time point post-depilation as indicated. **(D)** Percentage of cell types in the dorsal skin sample for each time point post-depilation as indicated. **(E)** Number of DE genes for each cell type along developmental time course. **(F)** Dot plot of ERBB ligands and receptors in each cell type. **(G)** Line chart of average expression of genes in the ERBB signaling pathway along developmental time course for the cells in the OB1 cluster. **(H)** Expression plot of *Bcl-2* family genes in all cells. The same UMAP plot in (**A**), colored by the relative expression (gray=low, blue=high).

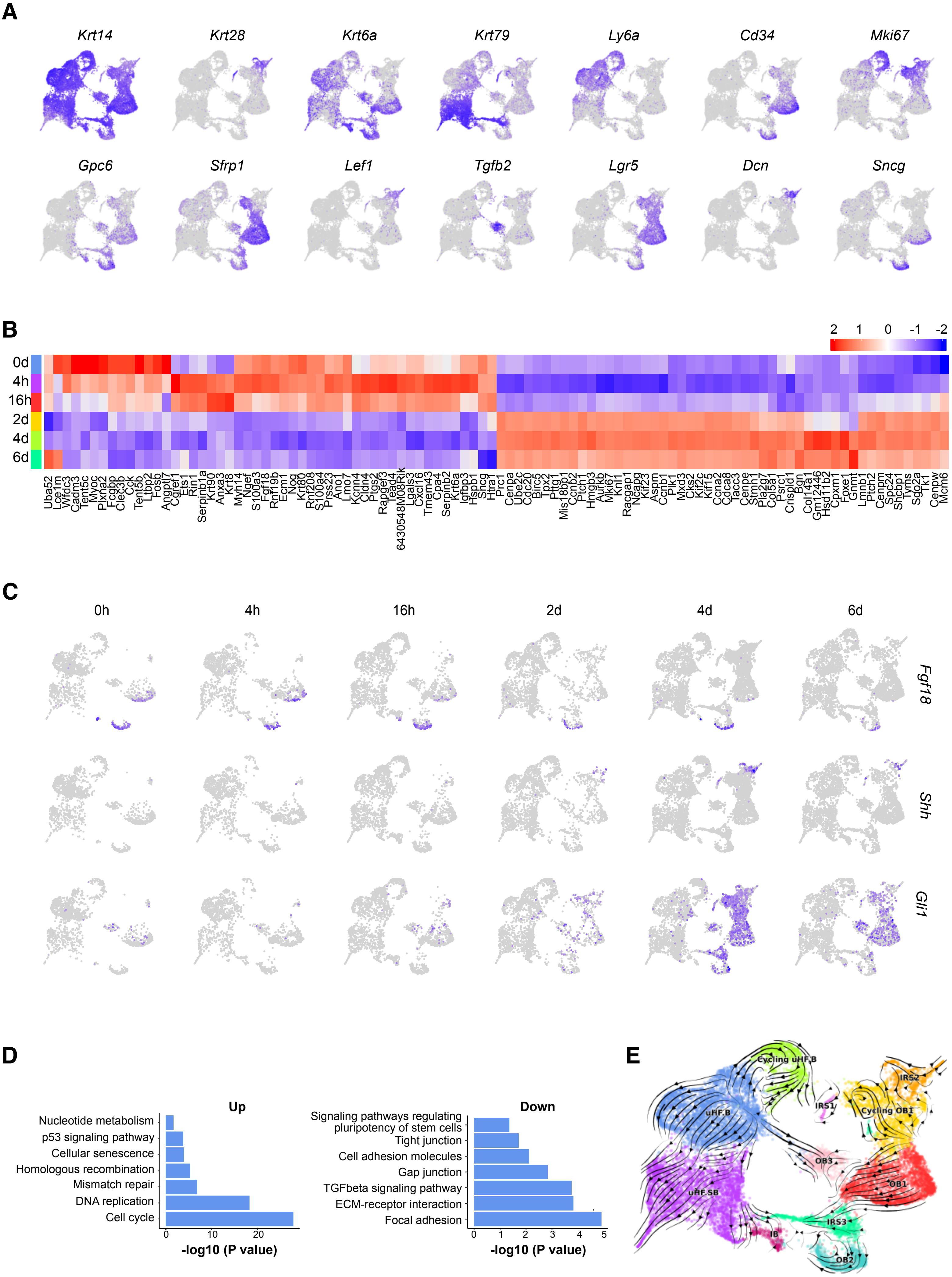
A
Krt14
Krt28
Krt6a
Krt79
Ly6a
Cd34
Mki67
Gpc6
Sfrp1
Lef1
Tgfb2
Lgr5
Dcn
Sncg
B
2 1 0 -1 -2
0d
4h
16h
2d
4d
6d
Uba52 Lce1m Wfdc3 Cadm3 Tent5c Myoc Plxna2 Fcgbp Clec3b Cck Tent5b Ltbp2 Fosb Angptl7 Cgref1 Ets1 Rin1 Serpinb1a Krt90 Anxa3 Krt8 Myh14 Ngef S100a3 Fgf18 Rnf19b Ecm1 Nog Krt80 Rnf208 S100a4 Prss23 Ano1 Lmo7 Kcnn4 Cldn4 Ptgs2 Rapgef3 Tead4 6430548M08Rik Lgals3 Cxcl16 Tmem43 Cpa4 Serpinb2 Krt6a Igfbp3 Hspb1 Sncg Htra1 Prc1 Cenpa Ube2c Cdc20 Birc5 Tpx2 Pttg1 Mis18bp1 Ccnb2 Ptch1 Hmgb3 Aurkb Mki67 Knl1 Racgap1 Ncapg Kif23 Aspm Ccnb1 Plk1 Mxd3 Cks2 Kif2c Kif15 Ccna2 Cdca8 Tacc3 Cenpe Stmn1 Pla2g7 Col5a1 Psrc1 Crispld1 Bgn Col14a1 Gm12446 Hsd11b2 Cpxm1 Foxe1 Gnmt Lmnb1 Ptch2 Cenpm Spc24 Shcbp1 Tyms Sgo2a Tk1 Cenpw Mcm6
C
0h
4h
16h
2d
4d
6d
Fgf18
Shh
Gli1
D
Up
Nucleotide metabolism
p53 signaling pathway
Cellular senescence
Homologous recombination
Mismatch repair
DNA replication
Cell cycle
0 10 20
-log10 (P value)
Down
Signaling pathways regulating pluripotency of stem cells
Tight junction
Cell adhesion molecules
Gap junction
TGFbeta signaling pathway
ECM-receptor interaction
Focal adhesion
0 1 2 3 4 5
-log10 (P value)
E
Cycling uHF.B
uHF.B
IRS1
IRS2
Cycling OB1
OB3
OB1
uHF.SB
IRS3
IB
OB2

Figure S8. Single-cell RNA-seq analysis of the dynamics of adult HF cells upon depilation-induced anagen

(A) Expression of selected genes in HF cells from the six samples. The same UMAP in Figure 5A, colored by the relative expression (gray=low, blue=high). **(B)** Heatmap of top 50 up and 50 downregulated DE genes in the cells of the OB1 cluster along the time course during hair regeneration. **(C)** Expression of Fgf18, Shh and Gli1 in HF cells from the six samples. The same UMAP plots in Figure 5D, colored by the relative expression (gray=low, blue=high). **(D)** Upregulated and downregulated KEGG pathway analysis of the DE genes in the cells of the OB1 cluster along the time course during hair reaeration. **(E)** RNA velocity plot of integrated HF cells of all samples during hair regeneration.

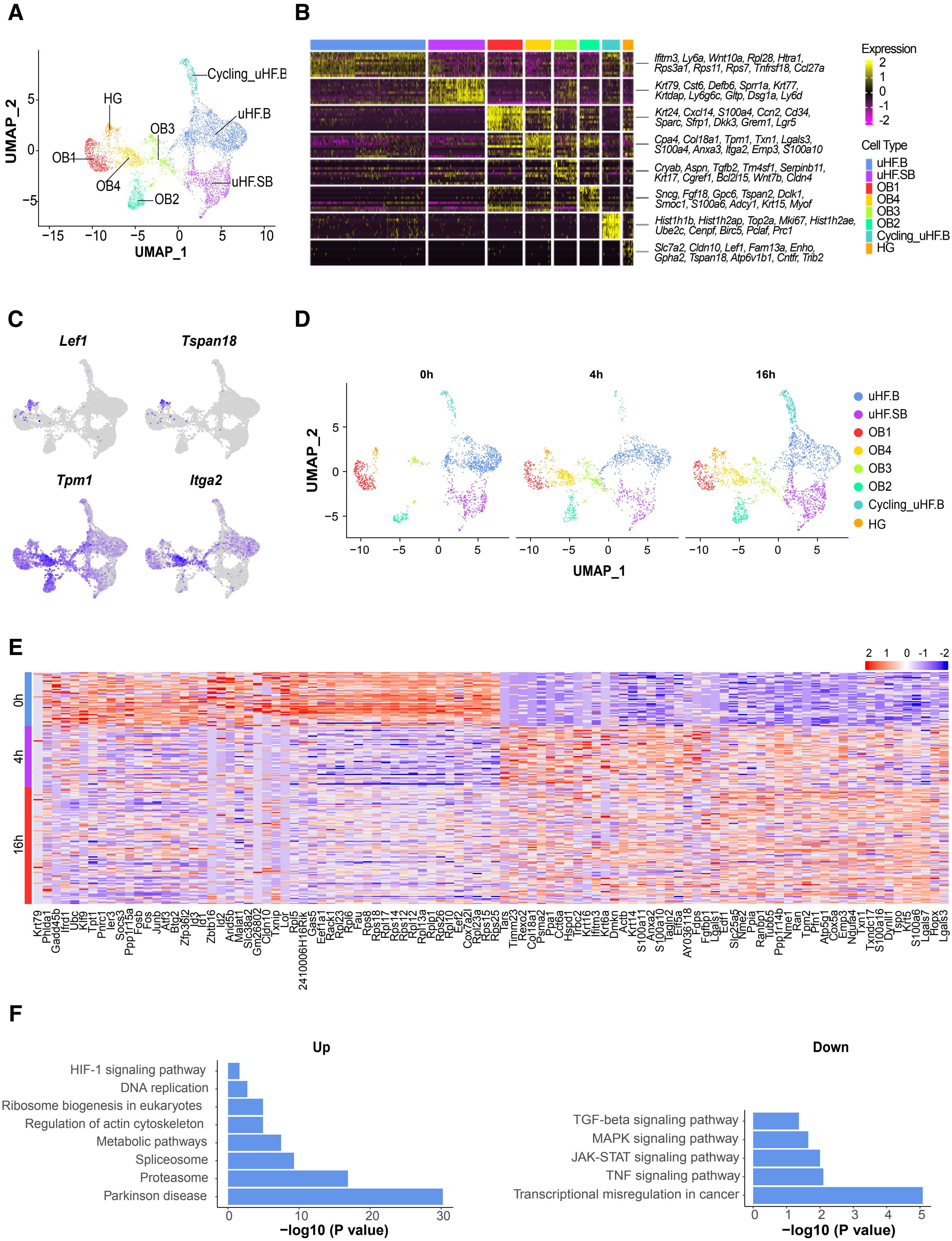
A
B
C
D
E
F
UMAP_2
UMAP_1
Cycling_uHF.B
HG
OB3
uHF.B
OB1
OB4
OB2
uHF.SB
Ifitm3, Ly6a, Wnt10a, Rpl28, Htra1, Rps3a1, Rps11, Rps7, Tnfrsf18, Ccl27a
Krt79, Cst6, Defb6, Sprr1a, Krt77, Krtdap, Ly6g6c, Gltp, Dsg1a, Ly6d
Krt24, Cxcl14, S100a4, Ccn2, Cd34, Sparc, Sfrp1, Dkk3, Grem1, Lgr5
Cpa4, Col18a1, Tpm1, Txn1, Lgals3, S100a4, Anxa3, Itga2, Emp3, S100a10
Cryab, Aspn, Tgfb2, Tm4sf1, Serpinb11, Krt17, Cgref1, Bcl2l15, Wnt7b, Cldn4
Sncg, Fgf18, Gpc6, Tspan2, Dclk1, Smoc1, S100a6, Adcy1, Krt15, Myof
Hist1h1b, Hist1h2ap, Top2a, Mki67, Hist1h2ae, Ube2c, Cenpf, Birc5, Pclaf, Prc1
Slc7a2, Cldn10, Lef1, Fam13a, Enho, Gpha2, Tspan18, Atp6v1b1, Cntfr, Trib2
Expression
Cell Type
Lef1
Tspan18
Tpm1
Itga2
0h
4h
16h
Up
HIF-1 signaling pathway
DNA replication
Ribosome biogenesis in eukaryotes
Regulation of actin cytoskeleton
Metabolic pathways
Spliceosome
Proteasome
Parkinson disease
−log10 (P value)
Down
TGF-beta signaling pathway
MAPK signaling pathway
JAK-STAT signaling pathway
TNF signaling pathway
Transcriptional misregulation in cancer

Figure S9. **Rapid responses of the HG and OB populations in gene expression program upon hair depilation.**

(A) UMAP visualization of HF cells in the murine dorsal skin across the three samples at 0, 4 and 16 h post depilation, colored according to the main cell classes. The plot was generated by integration of 1732, 1627 and 2342 HF cells for 0 h, 4 h and 16 h post depilation, respectively. **(B)** Heatmap of top 10 marker genes for each cell type. **(C)** Expression plots of marker genes of HG and OB4 cells for (A). The same UMAP in (**A**), colored by the relative expression (gray=low, blue=high). **(D)** UMAP visualization of all cells in the dorsal skin sample for each time point post-depilation as indicated. A distinct OB4 cluster appeared even at 4 h post depilation. **(E)** Heatmap of top 50 up and 50 downregulated genes of HG cells in the dorsal skin samples of 4 h and 16 h compared to 0 h. **(F)** Up and downregulated KEGG pathway analysis of the DE genes in HG cells in the dorsal skin samples of 4 h and 16 h compared to 0 h.

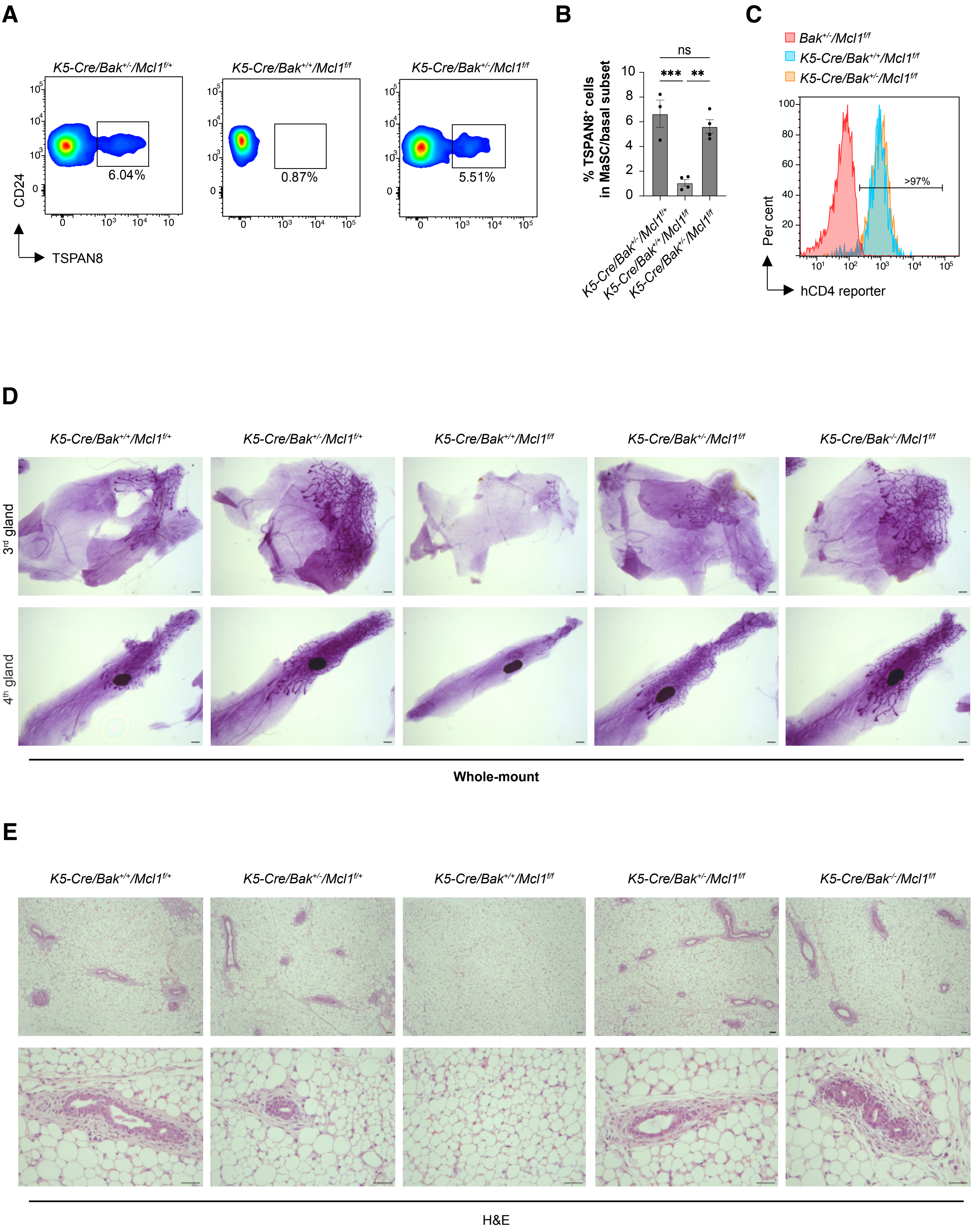
A
K5-Cre/Bak+/-/Mcl1f/+
K5-Cre/Bak+/+/Mcl1f/f
K5-Cre/Bak+/-/Mcl1f/f
6.04%
0.87%
5.51%
CD24
TSPAN8
B
% TSPAN8+ cells in MaSC/basal subset
ns
***
**
C
Bak+/-/Mcl1f/f
K5-Cre/Bak+/+/Mcl1f/f
K5-Cre/Bak+/-/Mcl1f/f
>97%
Per cent
hCD4 reporter
D
K5-Cre/Bak+/+/Mcl1f/+
K5-Cre/Bak+/-/Mcl1f/+
K5-Cre/Bak+/+/Mcl1f/f
K5-Cre/Bak+/-/Mcl1f/f
K5-Cre/Bak-/-/Mcl1f/f
3rd gland
4th gland
Whole-mount
E
K5-Cre/Bak+/+/Mcl1f/+
K5-Cre/Bak+/-/Mcl1f/+
K5-Cre/Bak+/+/Mcl1f/f
K5-Cre/Bak+/-/Mcl1f/f
K5-Cre/Bak-/-/Mcl1f/f
H&E

Figure S10: Deletion of a single *Bak* allele restores TSPAN8^{hi} quiescent stem cells and rescue the defects in *Mcl-1*-deficient mammary glands

(A, B) TSPAN8^{hi} quiescent stem cells are eliminated in *Mcl-1*-deficient mammary glands but restored by deletion of a single *Bak* allele. Representative FACS plots showing the TSPAN8^{hi} population in the basal compartment (Lin^{-}CD29^{hi}CD24^{+}) (A). Bar graph showing the percentage of cells in the basal population. Error bars represent mean ± SEM (B). Mammary glands from 10-12 weeks-old virgin females were processed and analysed. N=3 mice for each genotype. **(C)** Representative FACS plots demonstrating highly efficient recombination at the floxed *Mcl-1* allele. More than 97% of basal cells from *K5-cre/Bak*$^{+/+}$*/Mcl-1*$^{f/f}$ and *K5-cre/Bak*$^{+/-}$*/Mcl-1*$^{f/f}$ females expressed the hCD4 reporter but none could be detected in littermate controls. **(D, E)** Representative images of whole-mounted mammary glands (D) and H&E sections of mammary glands from 5-weeks-old females with indicated genotypes (E). n=3-4 mice for each genotype.

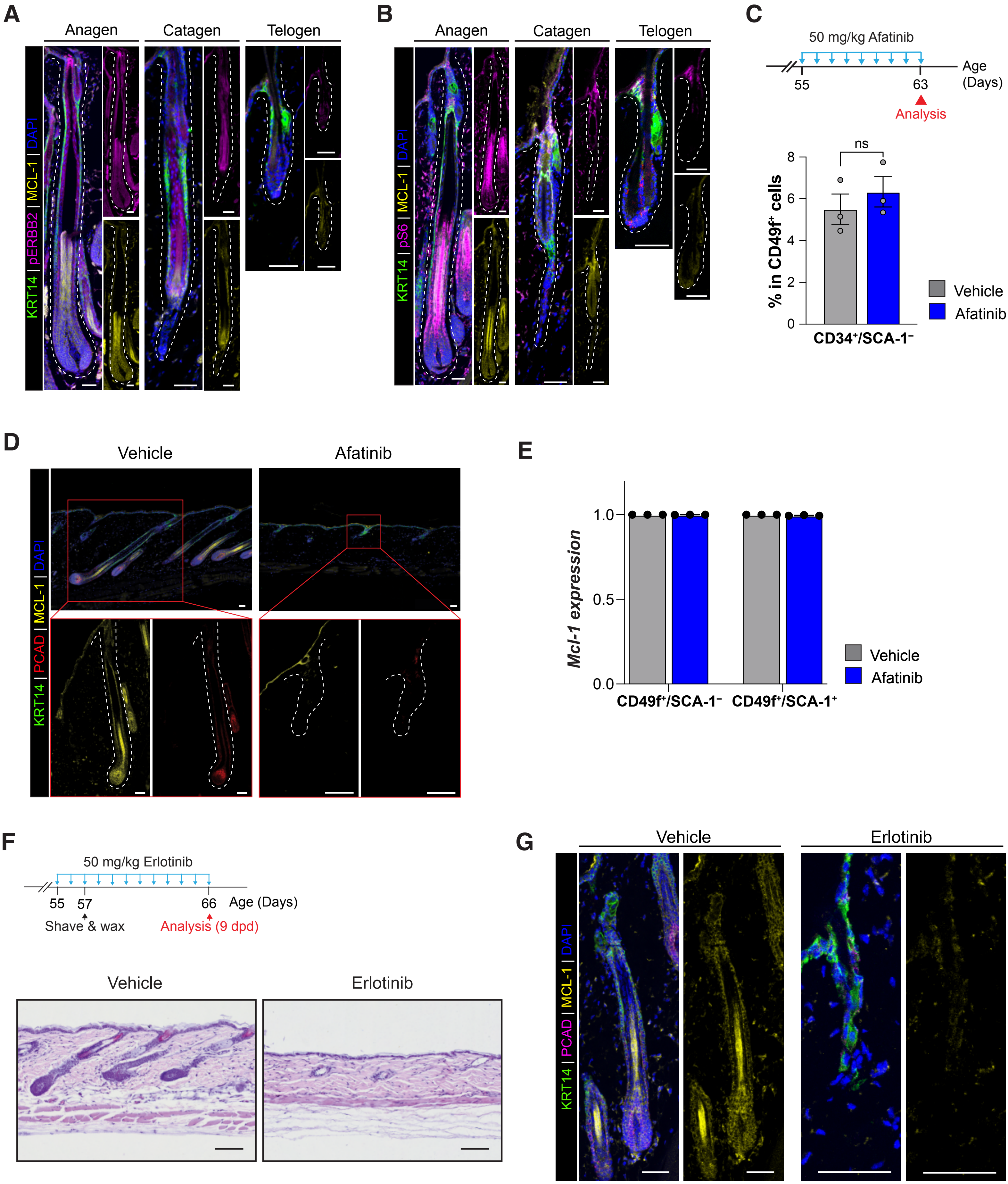

A
Anagen
Catagen
Telogen
KRT14 | pERBB2 | MCL-1 | DAPI
B
Anagen
Catagen
Telogen
KRT14 | pS6 | MCL-1 | DAPI
C
50 mg/kg Afatinib
55
63
Age (Days)
Analysis
ns
% in CD49f⁺ cells
8
6
4
2
0
CD34⁺/SCA-1⁻
Vehicle
Afatinib
D
Vehicle
Afatinib
KRT14 | PCAD | MCL-1 | DAPI
E
Mcl-1 expression
1.0
0.5
0.0
CD49f⁺/SCA-1⁻
CD49f⁺/SCA-1⁺
Vehicle
Afatinib
F
50 mg/kg Erlotinib
55
57
66
Age (Days)
Shave & wax
Analysis (9 dpd)
Vehicle
Erlotinib
G
Vehicle
Erlotinib
KRT14 | PCAD | MCL-1 | DAPI

Figure S11. Afatinib downregulates MCL-1 protein expression during hair regeneration

(A, B) Representative confocal images showing pERBB2 and MCL-1 (A) and pS6 and MCL-1 (B) expression in HFs at different stages of the HF cycle (anagen, catagen, and telogen). (*n*=3 mice, scale bars: 50 μm). KRT14 stains the basal skin layer, and DAPI serves as a nuclear counterstain. **(C)** Afatinib treatment does not affect the quiescent $CD34^{+}$ HFSC population. FVB/N mice at P55 received 50 mg/kg afatinib or vehicle daily for 9 days. FACS analysis of $CD49f^{+}/CD34^{+}/SCA\text{-}1^{-}$ HFSCs shows no significant difference between treatment groups. Data presented as mean ± SEM (*n*=3 mice per treatment, n.s. = non-significant; Student's t-test). **(D)** Afatinib reduces MCL-1 protein levels, impairing hair regeneration post-depilation. FVB/N mice at P55 were treated with 50 mg/kg afatinib or vehicle daily, with a portion of the dorsal skin shaved and waxed. Confocal analysis at 9 dpd shows reduced MCL-1 expression in afatinib-treated mice (*n*=9 per group, scale bars: 50 μm). KRT14 stains the basal skin layer, PCAD marks TACs, and DAPI is used as a nuclear counterstain. **(E)** qPCR analysis of Mcl-1 expression in FACS-sorted $CD49f^{+}/SCA\text{-}1^{-}$ (HF) and $CD49f^{+}/SCA\text{-}1^{+}$ (IFE) populations from afatinib-treated (*n*=3 per group). **(F, G)** Erlotinib impairs hair regeneration and reduces MCL-1 expression. FVB/N mice at P55 received 50 mg/kg erlotinib or vehicle daily, followed by depilation. H&E-stained sections of dorsal skin at 9 dpd (F) and confocal imaging of MCL-1, KRT14, and PCAD in HFs (G) show reduced MCL-1 expression in erlotinib-treated mice (*n*=3 per group, scale bars: 50 μm). DAPI serves as a nuclear counterstain.

SUPPLEMENTARY METHODS

Oligonucleotides for real-time PCR

mRNA	Sequence	Source
Mcl-1 set 1	(1) 5’-AGAGCGCTGGAGACCCTG-3’<br>(2) 5’-CTATCTTATTAGATATGCCAGACC-3’	Integrated DNA Technologies
Mcl-1 set 2	(1) 5’-AAGTCCTCGCCTGCGTCAGCA-3’<br>(2) 5’-GTTCAAGCCGATGACCGCGTTTCT-3’	Integrated DNA Technologies
Bim	(1) 5’-CGACAGTCTCAGGAGGAACC-3’<br>(2) 5’-CCTTCTCCATACCAGACGGA-3’	Integrated DNA Technologies
Bak	(1) 5’-CTTTGGCTACCGTCTGGC-3’<br>(2) 5’-CAACCGCCTCTCTGTGCGA-3’	Integrated DNA Technologies
Trp53	(1) 5’- TGGAAGACAGGCAGACTTTTC-3’<br>(2) 5’-GATGGTAAGGATAGGTCGGC-3’	Integrated DNA Technologies
B-Actin	(1) 5’-CACTGTCGAGTCGCGTCCA-3’<br>(2) 5’-CCACGATGGAGGGGAATACAG-3’	Integrated DNA Technologies
