# Supplementary material for: MCL‑1 safeguards activated hair follicle stem cells to enable adult hair regeneration
Source: Nat Commun. 2025 Mar 22;16:2829. doi: 10.1038/s41467-025-58150-5 (PMC11929845; doi:10.1038/s41467-025-58150-5)
Supplement: Supplementary file 2 — Reporting Summary [file 41467_2025_58150_MOESM2_ESM.pdf]

nature portfolio

Corresponding author(s): Nai Yang Fu & Hui San Chin

Last updated by author(s): 12 Feb 2025

Reporting Summary

Nature Portfolio wishes to improve the reproducibility of the work that we publish. This form provides structure for consistency and transparency in reporting. For further information on Nature Portfolio policies, see our Editorial Policies and the Editorial Policy Checklist.

Please do not complete any field with "not applicable" or n/a. Refer to the help text for what text to use if an item is not relevant to your study. For final submission: please carefully check your responses for accuracy; you will not be able to make changes later.

Statistics

For all statistical analyses, confirm that the following items are present in the figure legend, table legend, main text, or Methods section.

n/a	Confirmed	
☐	☒	The exact sample size (n) for each experimental group/condition, given as a discrete number and unit of measurement
☐	☒	A statement on whether measurements were taken from distinct samples or whether the same sample was measured repeatedly
☐	☒	The statistical test(s) used AND whether they are one- or two-sided<br>Only common tests should be described solely by name; describe more complex techniques in the Methods section.
☒	☐	A description of all covariates tested
☐	☒	A description of any assumptions or corrections, such as tests of normality and adjustment for multiple comparisons
☐	☒	A full description of the statistical parameters including central tendency (e.g. means) or other basic estimates (e.g. regression coefficient) AND variation (e.g. standard deviation) or associated estimates of uncertainty (e.g. confidence intervals)
☐	☒	For null hypothesis testing, the test statistic (e.g. F, t, r) with confidence intervals, effect sizes, degrees of freedom and P value noted<br>Give P values as exact values whenever suitable.
☒	☐	For Bayesian analysis, information on the choice of priors and Markov chain Monte Carlo settings
☒	☐	For hierarchical and complex designs, identification of the appropriate level for tests and full reporting of outcomes
☒	☐	Estimates of effect sizes (e.g. Cohen's d, Pearson's r), indicating how they were calculated

Our web collection on statistics for biologists contains articles on many of the points above.

Software and code

Policy information about availability of computer code

Data collection	Data collection method is described in the Method section. Software used:<br>GraphPad Prism 10 Software<br>ImageJ<br>Flowjo software (Treestar) v10.9.0<br>LASX Office 1.4.7 28921<br>Cell Ranger 6.1.2<br>Adobe illustrator 2024
Data analysis	Data analysis method is described in the Method section. Software used:<br>GraphPad Prism 10 Software<br>ImageJ<br>Flowjo software (Treestar) v10.9.0<br>LASX Office 1.4.7 28921<br>Cell Ranger 6.1.2<br>Adobe illustrator 2024

For manuscripts utilizing custom algorithms or software that are central to the research but not yet described in published literature, software must be made available to editors and reviewers. We strongly encourage code deposition in a community repository (e.g. GitHub). See the Nature Portfolio guidelines for submitting code & software for further information.

Data

Policy information about availability of data

All manuscripts must include a data availability statement. This statement should provide the following information, where applicable:

- Accession codes, unique identifiers, or web links for publicly available datasets
- A description of any restrictions on data availability
- For clinical datasets or third party data, please ensure that the statement adheres to our policy

The GEO accession number for the scRNA-seq data is: GSE256251 (https://www.ncbi.nlm.nih.gov/geo/query/acc.cgi?&acc=GSE256251)

Research involving human participants, their data, or biological material

Policy information about studies with [human participants or human data]. See also policy information about [sex, gender (identity/presentation), and sexual orientation] and [race, ethnicity and racism].

Reporting on sex and gender	N/A
Reporting on race, ethnicity, or other socially relevant groupings	N/A
Population characteristics	N/A
Recruitment	N/A
Ethics oversight	N/A

Note that full information on the approval of the study protocol must also be provided in the manuscript.

Field-specific reporting

Please select the one below that is the best fit for your research. If you are not sure, read the appropriate sections before making your selection.

☑ Life sciences ☐ Behavioural & social sciences ☐ Ecological, evolutionary & environmental sciences

For a reference copy of the document with all sections, see nature.com/documents/nr-reporting-summary-flat.pdf

Life sciences study design

All studies must disclose on these points even when the disclosure is negative.

Sample size	The sample size employed in this study was determined based on the minimum number required to attain statistical significance. The estimated sample size was n = 4-9 per group. For confocal image analysis, sample size was n=3-8 per group. For sc-RNA seq data, n=2 mice per time point (0 h, 4 h, 6 h, 2 d, 4 d, 6 d post-depilation).
Data exclusions	Mice that did not survive due to sickness / fighting wounds were exluded.
Replication	In vivo experimental findings were reproducible as shown across multiple animals over multiple surgical cohorts. In vitro experimental findings were independently reproduced at least 3 times unless specified. All attempts at replication were successful.
Randomization	Mice were not randomized because they were genetically identical within groups. The relevant experimental controls were used in each experiment as described in this paper.
Blinding	The investigators were aware of the genetic backgrounds and experimental treatments for all experiments.

Behavioural & social sciences study design

All studies must disclose on these points even when the disclosure is negative.

Study description	N/A
Research sample	N/A
Sampling strategy	N/A
Data collection	N/A
Timing	N/A
Data exclusions	N/A
Non-participation	N/A
Randomization	N/A

Ecological, evolutionary & environmental sciences study design

All studies must disclose on these points even when the disclosure is negative.

Study description	N/A
Research sample	N/A
Sampling strategy	N/A
Data collection	N/A
Timing and spatial scale	N/A
Data exclusions	N/A
Reproducibility	N/A
Randomization	N/A
Blinding	N/A

Did the study involve field work? ☐ Yes ☒ No

Field work, collection and transport

Field conditions	N/A
Location	N/A
Access & import/export	N/A
Disturbance	N/A

Reporting for specific materials, systems and methods

We require information from authors about some types of materials, experimental systems and methods used in many studies. Here, indicate whether each material, system or method listed is relevant to your study. If you are not sure if a list item applies to your research, read the appropriate section before selecting a response.

Materials & experimental systems

n/a	Involved in the study	
☐	☒	Antibodies
☒	☐	Eukaryotic cell lines
☒	☐	Palaeontology and archaeology
☐	☒	Animals and other organisms
☒	☐	Clinical data
☒	☐	Dual use research of concern
☒	☐	Plants

Methods

n/a	Involved in the study	
☒	☐	ChIP-seq
☐	☒	Flow cytometry
☒	☐	MRI-based neuroimaging

Antibodies

Antibodies used

The following antibodies for western blotting were used at indicated dilutions:
anti-MCL-1 (rabbit, clone D2W9E, 1:300), anti-BCL-XL (rabbit, clone 54H6, 1:200), anti-BIM (rabbit, clone C34C5, 1:100), anti-BAK (rabbit, clone D4E4, 1:100), anti-activated (i.e. cleaved) CASPASE-3 (rabbit, clone 5A1E, 1:100), anti-pHER2/pErbB2 (Y1221/1222) (rabbit, clone 6B12, 1:100), anti-P-S6 ribosomal protein (S240/244) (rabbit, clone D68F8, 1:100), anti-LEF1 (rabbit, clone C12A5, 1:100), anti-PCNA (mouse, clone PC10, 1:100), and anti-E-Cadherin (Rabbit, clone 24E10, 1:200) antibodies from Cell Signaling; anti-mouse P-CADHERIN (goat, clone AF761, 1:400) antibody from R&D systems; anti-mouse CD34 (rat, clone RAM34, 1:50) antibody from eBiosciences; anti-pan-cytokeratin (AE-13) (mouse, clone sc-57012, 1:300), anti-CLAUDIN-1 (mouse, clone A-9, 1:100), as well as anti-KRT15 (mouse, clone LHK15, 1:200) antibodies from Santa Cruz; anti-GATA3 (mouse, clone L50-823, 1:50) antibody from BD Pharmingen; anti-FILAG-GRIN antibody (rabbit, 1:100) from BioLegend; anti-DESMOPLAKIN (rabbit, 1:100) and anti-PLECTIN (rat, 1:100) antibodies from BiCell; anti-GFP (chicken, 1:300), anti-LORICRIN (rabbit, 1:100), anti-CD4 (Rabbit, EPR19514, 1:200), and anti-CD8 (rabbit, EPR21769, 1:200) antibodies from Abcam; anti-RFP antibody (rabbit, 1:200) from Rockland; anti-KRT14 (rat, 1:1000), anti-KRT-10 (rabbit, 1:1000) and anti-KRT6 (rat, 1:1000) antibodies from Chen Ting Lab.

The following commercially available antibodies for FACS analysis were used at indicated dilutionsL
APC/cy7 anti-mouse/rat CD29 (rat, clone HM1-1, Cat. no. 102226, 1:200), Pacific Blue anti-mouse CD24 (rat, clone M1/69, Cat. no. 101820, 1:200), eFluor 450 anti-mouse CD34 (rat, clone RAM34) antibody from eBioscience; PE-Cy7 anti-mouse SCA-1 (Ly-6A/E) (rat, clone D7), BUV395 anti-human CD49f (integrin 6 chain) (rat, clone GoH3), BV711 anti-human CD4 (mouse, L120 (RUO)), APC anti-mouse CD31 (rat, clone MEC13.3), APC anti-mouse CD45 (rat, clone 30-F11), APC anti-mouse TER119 (rat, clone TER119) antibodies from BD Pharmingen, PE anti-mouse Tspan8 (rat IgG2b clone no. 657909, Cat. no. FAB6524P, 1:75) and APC anti-mouse Tspan8 (Rat IgG2b clone no. 657909, Cat. no. FAB6524A, 1:75) from R&D Systems.

Validation

All antibodies utilized in this study are commercially available, with the exception of anti-KRT14, anti-KRT10 and anti-KRT6 antibodies from Chen Ting lab. These antibodies were previously published here (Lu et al., eLife, 2020; PMID: 31898934).

Eukaryotic cell lines

Policy information about cell lines and Sex and Gender in Research

Cell line source(s)	N/A
Authentication	N/A
Mycoplasma contamination	N/A
Commonly misidentified lines (See ICLAC register)	N/A

Palaeontology and Archaeology

Specimen provenance	N/A
Specimen deposition	N/A
Dating methods	N/A

☐ Tick this box to confirm that the raw and calibrated dates are available in the paper or in Supplementary Information.

Ethics oversight	N/A

Note that full information on the approval of the study protocol must also be provided in the manuscript.

Animals and other research organisms

Policy information about studies involving animals; ARRIVE guidelines recommended for reporting animal research, and Sex and Gender in Research

Laboratory animals	All mice included in this study was either of FVB/C57BL/6 mixed background or C57BL/6 background as indicated in our methods section. The sources of our mice is indicated in our methods section. All mice used in this study range from 5 days to 5 months of age depending on the experiments. new born pups were used for the isolation of primary keratinocytes.
Wild animals	No wild animals were used in this study.
Reporting on sex	Males were used for sc-RNA seq. Mixed genders were used for other experiments.
Field-collected samples	No field-collected samples were used in this study.
Ethics oversight	All animal experiments were carried out under the approval of and guidelines of the SingHealth Institute Animal Care and Use Committee, and WEHI institutional guidelines in approval by the WEHI Animal Ethics Committee.

Note that full information on the approval of the study protocol must also be provided in the manuscript.

Clinical data

Policy information about clinical studies

All manuscripts should comply with the ICMJE guidelines for publication of clinical research and a completed CONSORT checklist must be included with all submissions.

Clinical trial registration	N/A
Study protocol	N/A
Data collection	N/A
Outcomes	N/A

Dual use research of concern

Policy information about dual use research of concern

Hazards

Could the accidental, deliberate or reckless misuse of agents or technologies generated in the work, or the application of information presented in the manuscript, pose a threat to:

No | Yes
[X] [ ] Public health
[X] [ ] National security
[X] [ ] Crops and/or livestock
[X] [ ] Ecosystems
[X] [ ] Any other significant area

Experiments of concern

Does the work involve any of these experiments of concern:

No | Yes
[X] [ ] Demonstrate how to render a vaccine ineffective
[X] [ ] Confer resistance to therapeutically useful antibiotics or antiviral agents
[X] [ ] Enhance the virulence of a pathogen or render a nonpathogen virulent
[X] [ ] Increase transmissibility of a pathogen
[X] [ ] Alter the host range of a pathogen
[X] [ ] Enable evasion of diagnostic/detection modalities
[X] [ ] Enable the weaponization of a biological agent or toxin
[X] [ ] Any other potentially harmful combination of experiments and agents

Plants

Seed stocks	N/A
Novel plant genotypes	N/A
Authentication	N/A

ChIP-seq

Data deposition

[ ] Confirm that both raw and final processed data have been deposited in a public database such as GEO.
[ ] Confirm that you have deposited or provided access to graph files (e.g. BED files) for the called peaks.

Data access links<br>May remain private before publication.	N/A
Files in database submission	N/A
Genome browser session<br>(e.g. UCSC)	N/A

Methodology

Replicates	N/A
Sequencing depth	N/A
Antibodies	N/A
Peak calling parameters	N/A
Data quality	N/A

Software | N/A

Flow Cytometry

Plots

Confirm that:

☒ The axis labels state the marker and fluorochrome used (e.g. CD4-FITC).

☒ The axis scales are clearly visible. Include numbers along axes only for bottom left plot of group (a 'group' is an analysis of identical markers).

☒ All plots are contour plots with outliers or pseudocolor plots.

☒ A numerical value for number of cells or percentage (with statistics) is provided.

Methodology

Sample preparation	Dorsal skin of mice were subjected to enzymatic digestion by trypsin / collagenase / hyaluronidase. Details are included in the methods section
Instrument	LSRForetessa (Becton Dickinson)
Software	Flowjo v10.9.0 (Treestar)
Cell population abundance	150,000-250,000 events
Gating strategy	Live cells > CD31/Cd45/Ter119 negative > CD49f+ > CD34 / SCA-1

☐ Tick this box to confirm that a figure exemplifying the gating strategy is provided in the Supplementary Information.

Magnetic resonance imaging

Experimental design

Design type	N/A
Design specifications	N/A
Behavioral performance measures	N/A
Imaging type(s)	N/A
Field strength	N/A
Sequence & imaging parameters	N/A
Area of acquisition	N/A

Diffusion MRI ☐ Used ☐ Not used

Preprocessing

Preprocessing software	N/A
Normalization	N/A
Normalization template	N/A
Noise and artifact removal	N/A
Volume censoring	N/A

Statistical modeling & inference

Model type and settings	N/A
Effect(s) tested	N/A

Specify type of analysis: ☐ Whole brain ☐ ROI-based ☐ Both

Statistic type for inference
(See Eklund et al. 2016)

N/A

Correction

N/A

Models & analysis

n/a	Involved in the study
X	☐ Functional and/or effective connectivity
X	☐ Graph analysis
X	☐ Multivariate modeling or predictive analysis

Functional and/or effective connectivity

N/A

Graph analysis

N/A

Multivariate modeling and predictive analysis

N/A

This checklist template is licensed under a Creative Commons Attribution 4.0 International License, which permits use, sharing, adaptation, distribution and reproduction in any medium or format, as long as yo appropriate credit to the original author(s) and the source, provide a link to the Creative Commons license, and indicate if changes were made. The images or other third party material in this article are include the article's Creative Commons license, unless indicated otherwise in a credit line to the material. If material is not included in the article's Creative Commons license and your intended use is not permitted by statutory regulation or exceeds the permitted use, you will need to obtain permission directly from the copyright holder. To view a copy of this license, visit http://creativecommons.org/licenses/by/4.0/
